# A Pediatric Food Allergy Support Group Can Improve Parent and Physician Communication: Results of a Parent Survey

**DOI:** 10.1155/2012/168053

**Published:** 2011-10-10

**Authors:** Ashika Sharma, Tracy Prematta, Tracy Fausnight

**Affiliations:** Penn State Hershey Milton S. Hershey Medical Center, 500 University Drive, Hershey, PA 17033, USA

## Abstract

*Rationale*. We sought to evaluate the impact of having an allergist at a food allergy support group (FASG) on the relationship between parents and their child's allergist. *Methods*. Ninety-eight online surveys were sent to parents who attend a FASG affiliated with our institution. Responses were analyzed looking for reasons for attending the support group and comfort with having an allergist present at the meetings. The main objective of this study was to evaluate the impact of having an allergist at the food allergy support group on the relationship between parents and their child's allergist. *Results*. The FASG decreased anxiety about food allergies for 77.7% of those who responded. Most (71.4%) felt the FASG improved their child's quality of life. Greater than 90% felt comfortable having an allergist at the support group meeting, and 64.3% felt that talking to an allergist at the FASG made it easier to speak with their child's allergist. *Conclusions*. FASG meetings appear to be a good way for families of children with food allergies to learn more about food allergies, improve quality of life, and increase comfort in communicating with a child's allergist.

## 1. Introduction

Food allergies affect up to 6% of preschool and school aged children. While the only available therapy for these children is strict avoidance of the offending foods, accidental reactions are common and occur in up to 50% of food-allergic children despite their best efforts to avoid the offending foods [1]. Food allergies have a significant impact on quality of life. Families must be vigilant about food allergen avoidance in a variety of settings including home, restaurants, schools, camps, and social gatherings. Food allergic reactions are the leading cause of emergency department visits for anaphylaxis in the United States. The burden of avoidance and fear of an accidental exposure can increase anxiety and result in reduced quality of life [2]. Food allergy has been shown to lower general health perception, limit family activities, and have a significant emotional as well as economic impact on the parent of the food allergic child [3]. Poor communication between physicians and parents may be a factor that contributes to parental anxiety, as they do not feel that their concerns are adequately addressed. Thus, with the increasing prevalence of food allergy and the absence of a cure, communication between parents of food-allergic children and their physicians is crucial. There have been few studies done that have looked at the parent perceptions of a food allergy support group; however, none have looked at the parent's comfort with having an allergist present at the meetings and how this impacts their relationship with their child's own allergist. 

In 2006, Dr. Jenifer LeBovidge's group at the Children's Hospital in Boston, Massachusetts did an initial study on food allergy support groups and developed the Food Allergy Parent Questionnaire [4]. This tool was designed to evaluate both parental adjustment to a food allergy diagnosis and parental coping with children's food allergy. They concluded that the measure may be useful in screening for parental anxiety, perceived impact of food allergies, level of family support, and coping skills. In 2008, LeBovidge et al. designed another study (where both parents and children attended workshops) to evaluate a group intervention for children with food allergy and their parents [5]. The purpose of this study was to increase parent-perceived competence in coping with food allergy and to decrease the parent-perceived burden associated with food allergy. Parent and child evaluations of the workshop were favorable, and the results showed that parent-perceived competence in coping with food allergy increased significantly from preworkshop to postworkshop and followup, parent-perceived burden associated with food allergy decreased from preworkshop to followup. Another study, done by Gupta et al. studied focus groups which were held to obtain information for the development of validated survey instruments to assess food allergy knowledge, attitudes, and beliefs of parents, doctors, and the general public [6]. In this relatively small study, it was concluded that the quality of life for children with food allergy and their families is significantly affected due to gaps in physician knowledge and public knowledge about food allergies. Results showed that parents of food allergy had solid fundamental knowledge but had concerns about primary care physicians' knowledge of food allergy, diagnostic approaches, and treatment practices. Physicians had good basic knowledge of food allergy but differed in their approach to diagnosis and advice about feeding. The general public had wide variation in knowledge about food allergy with many misconceptions of key concepts related to prevalence, definition, and triggers of food allergy. Parents expressed concern about effect of food allergy on quality of life and the associated anxiety about keeping their children safe. Parents also reported frustration in receiving a timely diagnosis and felt that physicians of different specialties provided conflicting guidance in the diagnosis and treatment of food allergy. A novel finding was the mothers' assertions that their child's food allergy caused them to stop working outside the home. It was difficult for many mothers to entrust others with the care of their child. 

The support group at our institution was designed to provide practical and emotional support that might better enable parents to handle the stress of their children's food allergies. The purpose of our study was to evaluate the impact of a food allergy support group (FASG) at our institution on quality of life and the relationship between parents and their child's allergist. We sought to understand how the interaction between parents and the allergist at the support group meetings affects the relationship between parents and their child's own allergist. This information would allow us to improve the support group experience in the future.

## 2. Methods

The support group at our institution is listed on the Food Allergy and Anaphylaxis Network (FAAN) website, and therefore an open support group. Patients and families do not need to be seen at our institution in order to attend group meetings or social activities. The meetings are held one evening a month at our institution. The participants that attend the FASG include about 50% parents of children at our institution, and about 50% parents from outside the institution. After IRB approval, survey consent forms were sent by email to all 98 support group members (all over 18 yrs of age). The email contained a link to the 30-question online survey. Completion of the survey implied voluntary consent to participate in the research study. The consent form described the purpose of the research (i.e., to improve future allergy support group meetings) as well as a statement of confidentiality. The participants had subsequently met with their child's allergist when they answered the questionnaire. Our 30-question survey was designed with a few ideas in mind: to uncover reasons for attending the support group, gage parent satisfaction with the FASG, and comfort level with having an allergist present at the meetings. The main objective of this study was to evaluate the impact of having an allergist at the food allergy support group on the relationship between parents and their child's allergist. The first set of questions is information about the person completing the survey, including their relationship to the child. The next set of questions were about the child with food allergies including type of food allergies, type of allergic reactions the child has experienced, length of time since diagnosis, and length of time seeing an allergy specialist. The next few questions were to get a sense of the reasons why the participants had joined the support group and how many meetings they had attended. We also asked about participants' level of anxiety and level of comfort before and after attending the food allergy support group, and before and after meeting with an allergist. We also asked about comfort having an allergist at the support group meetings, emotional support, and comfort with food allergies before and after the meetings. We asked questions regarding the support group's impact on coping with managing allergies at school). Responses were analyzed looking for reasons for attending the support group, parent satisfaction, and comfort with having an allergist present at the meetings.

## 3. Results

**Demographics**

A total of 29 surveys (29.6%) were completed. All respondents were mothers of children with food allergies, and 28 classified themselves as Caucasian not of Hispanic origin. 23 respondents had a college education or higher. Children's ages ranged from 1 to 11 years old, and were equally split between males and females. 26 had peanut allergy, 12 had a tree nut allergy, 15 had an egg allergy, and 15 had milk allergy. Almost 90% of children had experienced rash/hives, and 60% of the children had experienced a severe allergic reaction (vomiting, abdominal pain, difficulty breathing, face or lip swelling). 28 of the 29 respondents stated that it had been >12 months since their child was diagnosed with a food allergy. Ten of the respondents starting seeing an allergist less than a month after diagnosis; 15 of respondents did so within 1–6 months after diagnosis. All 29 respondents felt comfortable speaking to their child's allergist.

**Reasons for Joining**

**Figure 1:**
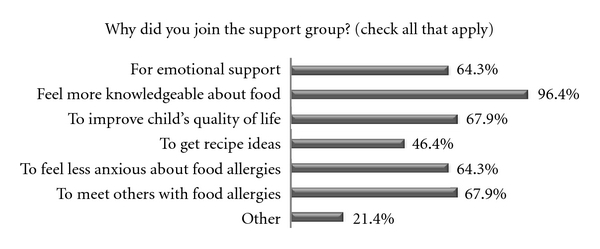


Fourteen respondents were referred to the support group by their own allergist. The number one reason for joining the FASG was to “feel more knowledgeable about food allergies”. Other reasons are shown in Figure 1 (see below).

**Anxiety**

The FASG decreased anxiety about food allergies for 77.7% of those who responded. Results showed that after parents attended support group meetings, almost 78% felt “very comfortable” caring for their child's food allergies, compared to only 3.7% that felt very comfortable prior to attending the FASG meetings.

**Quality of Life**

Most respondents (71.4%) felt the FASG improved their child's quality of life. 64.3% of respondents felt the FASG helped them manage allergies at family activities.

**Relationship between Parents and Allergist**

Twenty-eight out of twenty-nine respondents felt comfortable having an allergist at the support group meeting, and 64.3% felt that talking to an allergist at the FASG made it easier to speak with their child's allergist. Many of the respondents (77.8%) said they would continue to attend the support group meetings.

## 4. Discussion

As discussed earlier, there have been few studies done that have looked at the parent perceptions of a food allergy support group. Similar to other studies done by LeBovidge and Gupta, our study shows that a FASG has a positive effect on its members and provides many benefits including providing advice on food allergy risks and safety procedures, providing help in managing and coping with food allergies, and helping to decrease anxiety and increase comfort in dealing with food allergies.

However, none of these prior studies have looked at the parent's comfort with having an allergist present at the meetings and how this affects the relationship they have with their child's own allergist. This is the first study which, to our knowledge, has looked at the role of the physician in a FASG. From our results, it appears that having an allergist present at the meeting is a positive feature. Having the allergist available improved the parents' ability to communicate with their own allergist. Unfortunately, the availability of an allergist to be able to attend food allergy support group meetings is unclear. 

These findings provided preliminary support for the effectiveness and feasibility of a group intervention for the parents of children with food allergy. FASG meetings appear to be a good way for families of children with food allergies to learn more about food allergies and become comfortable with their child's diagnosis. Parents who feel more competent in managing their child's medical condition can also help their child develop better coping skills. Participating in FASG meetings can improve quality of life and if there is an allergist present, they can also increase comfort in communicating with a child's own allergist.

## References

[1] Nowak-Wegrzyn A, Conover-Walker MK, Wood RA (2001). Food-allergic reactions in schools and preschools. *Archives of Pediatrics and Adolescent Medicine*.

[2] Sicherer SH, Noone SA, Muñoz-Furlong A (2001). The impact of childhood food allergy on quality of life. *Annals of Allergy, Asthma and Immunology*.

[3] Marklund B, Ahlstedt S, Nordström G (2007). Food hypersensitivity and quality of life. *Current Opinion in Allergy and Clinical Immunology*.

[4] LeBovidge JS, Stone KD, Twarog FJ (2006). Development of a preliminary questionnaire to assess parental response to children’s food allergies. *Annals of Allergy, Asthma and Immunology*.

[5] LeBovidge JS, Timmons K, Rich C (2008). Evaluation of a group intervention for children with food allergy and their parents. *Annals of Allergy, Asthma and Immunology*.

[6] Gupta RS, Kim JS, Barnathan JA, Amsden LB, Tummala LS, Holl JL (2008). Food allergy knowledge, attitudes and beliefs: focus groups of parents, physicians and the general public. *BMC Pediatrics*.

